# Hepatic cancer stem cell marker granulin-epithelin precursor and β-catenin expression associate with recurrence in hepatocellular carcinoma

**DOI:** 10.18632/oncotarget.7803

**Published:** 2016-03-01

**Authors:** Phyllis F.Y. Cheung, Tan To Cheung, Chi Wai Yip, Linda W.C. Ng, Sze Wai Fung, Chung Mau Lo, Sheung Tat Fan, Siu Tim Cheung

**Affiliations:** ^1^ Department of Surgery, The Chinese University of Hong Kong, Hong Kong, China; ^2^ Department of Anatomical and Cellular Pathology, The Chinese University of Hong Kong, Hong Kong, China; ^3^ Department of Surgery, The University of Hong Kong, Hong Kong, China; ^4^ Department of Surgery, Queen Mary Hospital, Hong Kong, China; ^5^ School of Biomedical Sciences, The University of Hong Kong, Hong Kong, China; ^6^ Li Ka Shing Institute of Health Sciences, The Chinese University of Hong Kong, Hong Kong, China

**Keywords:** β-catenin, cancer stem cells, granulin-epithelin precursor, hepatocellular carcinoma

## Abstract

Granulin-epithelin precursor (GEP) has been demonstrated to confer enhanced cancer stem-like cell properties in hepatocellular carcinoma (HCC) cell line models in our previous studies. Here, we aimed to examine the GEP-expressing cells in relation to the stem cell related molecules and stem-like cell properties in the prospective HCC clinical cohort. GEP protein levels were significantly higher in HCCs than the paralleled non-tumor liver tissues, and associated with venous infiltration. GEP^high^ cells isolated from clinical HCC samples exhibited higher levels of stem cell marker CD133, pluripotency-associated signaling molecules β-catenin, Oct4, SOX2, Nanog, and chemodrug transporter ABCB5. In addition, GEP^high^ cells possessed preferential ability to form colonies and spheroids, and enhanced *in vivo* tumor-initiating ability while their xenografts were able to be serially subpassaged into secondary mouse recipients. Expression levels of GEP and pluripotency-associated genes were further examined in the retrospective HCC cohort and demonstrated significant correlation of GEP with β-catenin. Notably, HCC patients with high GEP and β-catenin levels demonstrated poor recurrence-free survival. In summary, GEP-positive HCC cells directly isolated from clinical specimens showed β-catenin elevation and cancer stem-like cell properties.

## INTRODUCTION

Tumor heterogeneity has long been explained by clonal evolution of tumor cells resulting from progressive accumulation of numerous genetic or epigenetic changes [1, 2]. However, recent studies have suggested that such heterogeneity results from the hierarchical organization of tumor cells with a subset of cells called cancer stem cells (CSCs) lying at the apex of the hierarchy [3]. The proposed concept of cancer as an abnormal stem cell disease was based on the similarities of cancer cells and normal stem cells to self-renew and produce heterogeneous progenies [4]. CSCs are highly tumorigenic, resistant to conventional therapies, and are responsible for tumor relapse [5]. The presence of CSCs has been reported in diverse malignancies including breast [6], colon [7] and liver cancers [8, 9]. Although advances have been made in hepatic CSC identification, precise origin of these cells and the underlying regulatory mechanism remain unclear.

Granulin-epithelin precursor (GEP) is a pluripotent growth factor regulating early fetal development, adult tissue repair, inflammation and tumorigenesis [10]. Our group has demonstrated over-expression and tumorigenic role of GEP in HCC [11–15]. GEP blockage using antibody effectively sensitized HCC cells to chemotherapeutic agents and inhibited the growth of established HCC xenografts [14, 16]. Recently, we revealed that GEP was a hepatic oncofetal protein regulating hepatic cancer stem cell (CSC) properties [17] and rendering HCC cells resistant to NK cytotoxic activity [18]. Interestingly, GEP was shown to regulate the expression of pluripotency-associated signalling molecules β-catenin, Oct4, Nanog and SOX2 in HCC cell lines [17].

Wnt signaling pathway is crucial for self-renewal and proliferation of stem cells in various tissues [19]. β-catenin protein, encoded by the *CTNNB1* gene, is the central player in canonical Wnt pathway. β-catenin can be found in several cellular compartments, including the inner plasma membrane, cytoplasm and nucleus [20]. Upon activation, cytoplasmic β-catenin accumulates and translocates into nucleus, where it forms an active complex containing T-cell factor/lymphoid enhancer factor transcription factors that induces the expression of its target genes [21]. Wnt pathway is activated in various types of cancer constitutively, leading to cell reprogramming and stem-like phenotype [22, 23]. β-catenin activation has been observed in different hepatic CSC subpopulations such as CD133+, EpCAM+, and GEP+ cells and might play role in maintaining the hepatic CSC features [9, 17, 24, 25]. Aberrant Wnt/β-catenin signaling by mutational and non-mutational events is observed in around one third of HCCs, implying the significance of this pathway in hepatocarcinogenesis [26]. In fact, deregulation of Wnt/β-catenin signaling is involved in early hepatocarcinogenesis and is associated with aggressive features of HCC, due to its role in cell survival, proliferation, migration and invasion [21, 27].

In this study, we characterized the CSC properties of GEP-expressing cells in the prospective HCC clinical cohort, and elucidated the underlying molecular mechanism with respect to the stem cell-related signaling molecules. These have been further substantiated in the retrospective HCC cohort.

## RESULTS

### GEP positive HCC cells in clinical specimens exhibit cancer stem cell phenotype

HCC and paralleled non-tumor liver tissues from 90 patients were freshly collected. After single cell isolation, only HCCs with high cell viability (viability ≥ 70%) were subject to subsequent phenotypic characterization and functional assays. A total of 42 HCCs (47%) generated cells with high viability. Importantly, HCCs with high cell viability were significantly associated with presence of venous infiltration, poorly-differentiated tumors and high AFP levels (Supplementary Table S1). Therefore, the more aggressive subset of HCCs were able to tolerate the single-cell isolation procedure for subsequent functional experiments.

**Figure 1:**
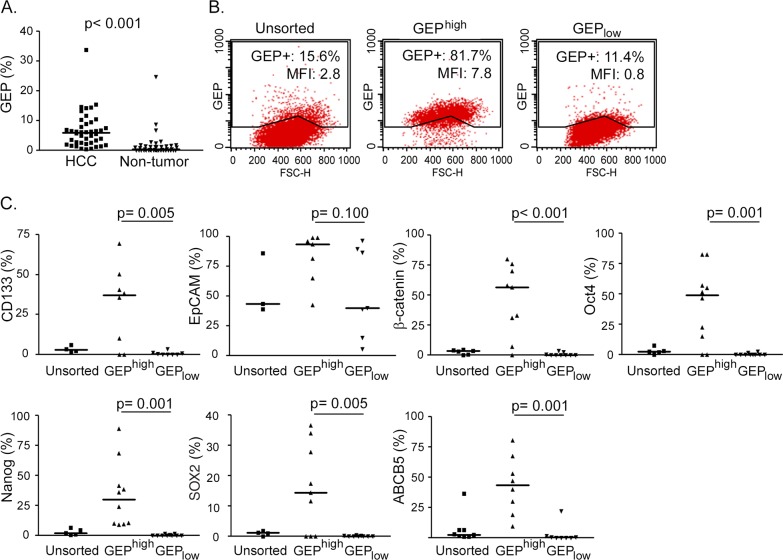
GEP positive HCC cells express stem cell related molecules Fresh HCC and paralleled non-tumor liver tissues were collected. After enzymatic digestion, cell viabilities were assessed by trypan blue staining, and only cases with high cell viability (viability ≥ 70%) (n = 42/90, 47%) were subject to subsequent characterization. **A.** Cells isolated from fresh HCC and non-tumor tissues were stained for total cellular GEP and analyzed by flow cytometry (n = 42). **B.** Cells were sorted according to cell surface GEP. Sorted GEP^high^ and GEP_low_ HCC cells were then permeabilized and stained for cellular GEP using anti-GEP antibody recognizing different epitope from that of the sorting antibody, and analyzed by flow cytometry. Percentage of GEP+ cells and mean fluorescence intensity (MFI) of the unsorted and sorted populations were shown. **C.** Sorted GEP^high^ and GEP_low_ cells and unsorted control were stained for hepatic surface CSC markers CD133 and EpCAM, pluripotency-associated signaling molecules β-catenin, Oct4, Nanog and SOX2, and drug transporter ABCB5, and analyzed by flow cytometry (n > 7 for each marker). The lines in scatter plots indicated the median values.

Previously, our group has demonstrated by RT-qPCR and immunohistochemistry that GEP levels were significantly elevated in HCC when compared with paralleled adjacent non-tumor liver tissues [11, 13]. Here, the GEP protein expressions have been quantified by flow cytometry in the 42 HCCs with high cell viability. GEP expression ranged from 0.4 to 34.3% (mean, 6.8%; median, 6.1%) (GEP+, %) in HCC tumor tissues, and was significantly higher than their paired adjacent non-tumor liver counterparts (p < 0.001, n = 42) (Figure 1A). In addition, GEP levels were positively associated with venous infiltration (p = 0.030) (Table 1). The result corroborated with our previous observation that strong GEP expression by immunohistochemistry was associated with venous infiltration [11].

**Table 1:** HCC clinico-pathological features in relation to GEP levels

Clinico-pathological variables	GEP^		*P* value
	Low	High	
**Age**			
Young (≤60)	15	7	0.827
Elderly (>60)	13	7	
**Venous infiltration**			
Absent	11	1	**0.030**
Present	17	13	
**HBV status**			
Negative for HBsAg	6	4	0.608
Positive for HBsAg	22	10	
**Cellular differentiation (Edmondson-Steiner grade)**			
Well-differentiated	18	8	0.750
Poorly-differentiated	9	5	
**Tumor stage (version UICC7)**			
Early stage	15	5	0.418
Late stage	10	6	
**Tumor size**			
Small (≤5cm)	7	3	0.798
Large (>5cm)	21	11	
**Serum AFP level**			
Low (≤400 ng/ml)	13	8	0.513
High (>400 ng/ml)	15	6	
**Number of tumor nodules**			
Single	18	8	0.653
Multiple (≥2)	10	6	

^ The cutoff for GEP expression levels was 6.92 (% of GEP+ cells), which was determined by maximizing the Youden index for the prediction of 1-year recurrence-free survival.

GEP was shown to co-express with stem cell markers in cell models [17]. Here, GEP^high^ and GEP_low_ subpopulations were isolated from freshly resected HCC specimens. GEP is an autocrine and paracrine growth factor detectable both on the cell surface and intracellularly. The cells were sorted according to cell surface GEP, and not permeabilized for intracellular GEP, in order to maintain the cell viability for subsequent functional assays. Thus all cells in the GEP^high^ subpopulation were positive for cell surface GEP. For the post-sorting analysis using a different antibody for detection, the cells were permeabilized and stained for intracellular GEP. Approximately 80-85% of GEP^high^ cells expressed GEP, while about 10% was detected in the GEP_low_ subpopulation (Figure 1B).

The sorted cells were examined for their expression of stem cell markers using flow cytometry. GEP^high^ cells isolated from clinical HCCs expressed significantly higher levels of hepatic CSC markers CD133, pluripotency-associated signaling molecules β-catenin, Oct4, Nanog, SOX2 and ABC drug transporter ABCB5, than their GEP_low_ counterparts (Figure 1C). GEP^high^ cells also expressed higher level of another hepatic marker EpCAM than GEP_low_ cells, although statistical significant difference has not been reached. The data is consistent with our previous findings that GEP preferentially co-expressed with stem cell markers in cell models [17].

### GEP^high^ cells possess CSC properties *in vitro*

**Figure 2:**
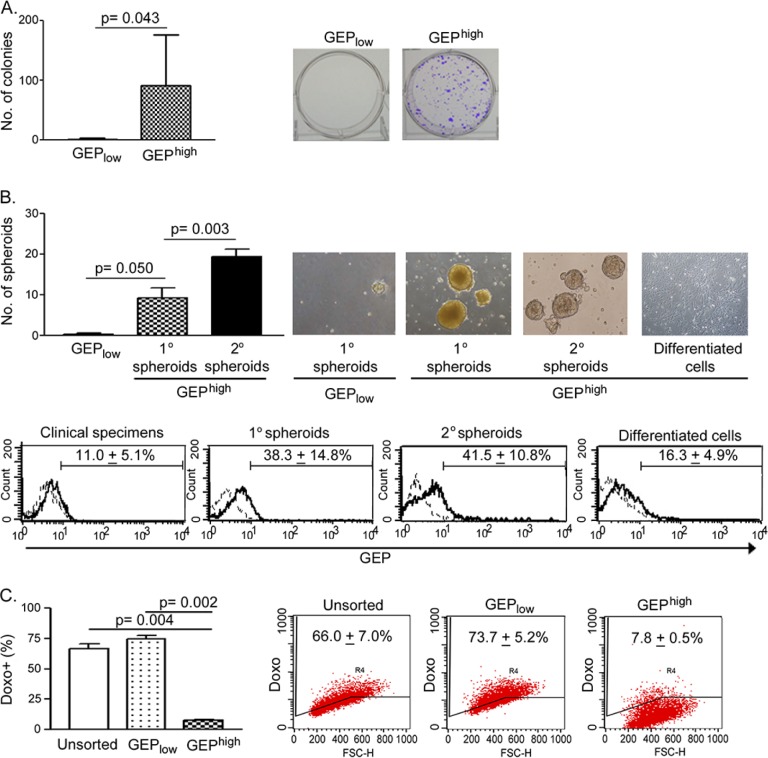
GEP^high^ cells possess CSC properties *in vitro* **A.** Sorted GEP^high^ cells isolated from freshly resected HCC were able to form more colonies than GEP_low_ cells (n = 6). In brief, 1000 cells of each freshly sorted subpopulation were seeded onto 6-well plate and allowed to grow for a month. **B.** Upper panel: GEP^high^ cells, but not GEP_low_ cells, isolated from freshly resected tumors were able to generate primary (1°) spheroids (n = 3). 1° spheroids were collected and dissociated, and 1000 disaggregated cells were allowed to grow for 1 month for generating secondary (2°) spheroids (n = 3). GEP^high^ cell-derived, but not GEP_low_ cells-derived 1° spheroids, were able to generate 2° spheroids. Following induced differentiation, disaggregated cells generated from GEP^high^ cell-derived 2° spheroids differentiated and grew as adherent cells. Lower panel: Flow cytometric analysis showed the enrichment of GEP expression in mechanically dissociated GEP^high^ cells-derived spheroids as compared to the original resected tumors (clinical specimens) and the differentiated adherent counterpart. Briefly, 1000 cells of each freshly sorted subpopulation were seeded into ultra-low attachment 24-well plate, and allowed to grow for 1 month to generate spheroids. **C.** After exposure to doxorubicin (0.5μg/ml) for 24h, GEP^high^ cells retained significantly less doxorubicin than GEP_low_ cells and unsorted control (n = 3). Data are expressed as mean percentage + SD.

To determine whether GEP^high^ cells were enriched for CSCs, we compared the tumorigenic potential and stem cell properties of GEP^high^ cells with their counterparts *in vitro*. Clonogenicity of the cells was assessed by colony formation assay. GEP^high^ cells were able to form significantly more colonies compared with GEP_low_ cells (n = 6) (Figure 2A).

To assess the self-renewal ability, GEP^high^ and GEP_low_ cells were allowed to grow as spheroids. Significantly greater number of spheroids were generated from GEP^high^ cells than GEP_low_ counterpart (n = 3) (Figure 2B, 1° spheroids). Moreover, when GEP^high^ cell-derived 1° spheroids were dissociated into desegregated cells, these cells could form second generations of spheroids (Figure 2B, 2° spheroids), which were clonally expanded for at least 10 more generations thereafter. The results therefore demonstrated the unlimited growth potential and self-renewal ability of GEP^high^ cells. Besides, it is noteworthy that GEP levels in the spheroids were higher than those in original resected tumors (Figure 2B, lower panel), implying that GEP was enriched in the spheroids and might play important role in maintaining the stemness of cells.

To determine the differentiation potential of these GEP-expressing cells, GEP^high^ cell-derived 2° spheroids were dissociated and cultivated in the presence of serum but without growth factor supplements on adherent plate. After few days, the cells attached onto the plates and grew as adherent cells (Figure 2B, differentiated cells). As compared with spheroids, the differentiated and adherent counterparts expressed lower GEP level following the induced *in vitro* differentiation (Figure 2B, lower panel).

To examine the role of GEP in chemoresistance, cells were incubated with doxorubicin and assessed for intracellular drug accumulation. GEP^high^ subpopulations demonstrated significantly lower doxorubicin retention when compared with the unsorted control and GEP_low_ subpopulations (n = 3) (Figure 2C).

### GEP^high^ cells are more tumorigenic *in vivo*

**Figure 3:**
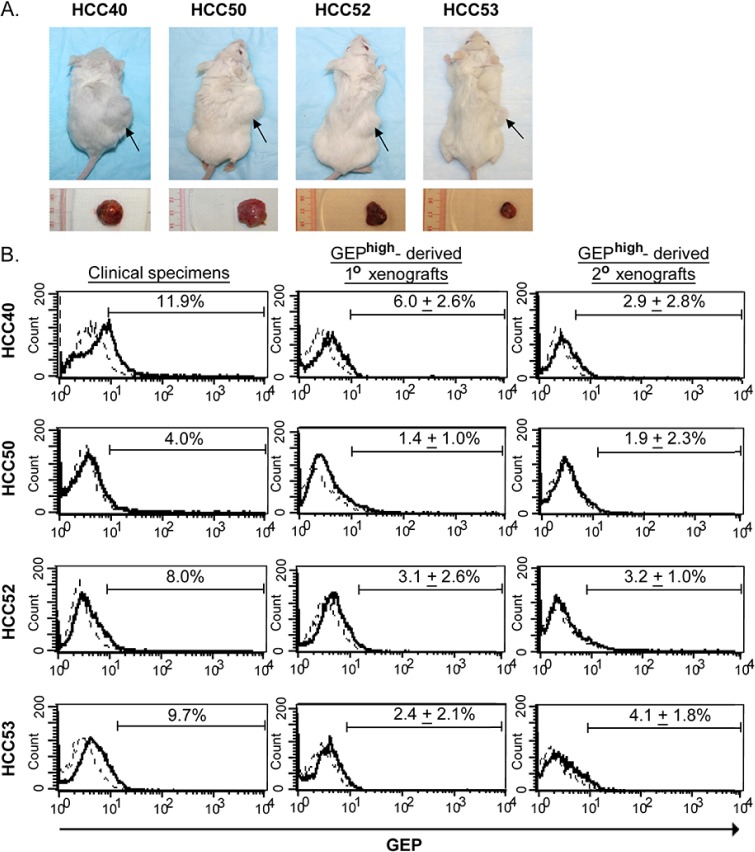
GEP^high^ cells possess CSC properties *in vivo.* GEP^high^ and GEP_low_ cells were sorted from freshly resected tumors of HCC patients and injected into NOD/SCID mice. **A.** The upper panel shows the NOD/SCID mice injected subcutaneously with 1× 10^4^ GEP^high^ (right flank) and GEP_low_ (left flank) cells at 8-14 weeks after inoculation. Arrow indicated the site of tumor formation at the right flank of mice. The lower panel shows the corresponding subcutaneous tumors derived from GEP^high^ cells. **B.** Flow cytometric analysis showed the total cellular GEP expression in mechanically dissociated GEP^high^ cells derived-xenograft tumors (1° and 2°) as compared to the original clinical specimens. In brief, to demonstrate the *in vivo* self-renewal ability of the cells, GEP^high^ and GEP_low_ subpopulations were sorted from xenografts growing from initial inoculation (1° xenografts) and then transplanted into secondary mouse recipients. GEP^high^, but not GEP_low_ subpopulations, were able to generate secondary (2°) xenograft tumors. Data are expressed as mean percentage + SD.

*In vivo* tumorigenicity assay using GEP^high^ and GEP_low_ subpopulations isolated from HCC clinical specimens were examined in immunocompromised NOD/SCID mice. Cells isolated from 4 out of 11 HCC tumor specimens (36%) could generate xenograft tumors. Among these 4 cases, almost all mice injected with GEP^high^ cells could generate visible tumors before 15 weeks post-injection (Figure 3A and Table 2). As few as 1×10^3^ GEP^high^ cells were able to generate visible tumors 8-15 weeks after injection. On the contrary, no tumor could be observed from GEP_low_ cells at all doses (Figure 3A and Table 2). Our result demonstrated that the cells capable of initiating HCC were highly enriched in GEP^high^ subpopulation.

**Table 2:** *In vivo* tumorigenicity of sorted GEP^high^ and GEP_low_ subpopulations from clinical HCC specimens

Case no.	Sorted cells^	Cell no. injected	Tumor incidence	Latency (weeks)
**40**	**GEP^high^**	1×10^5	3/3	4, 5, 5
		1×10^4	3/3	5, 5, 5
		1×10^3	4/4	8, 11, 15, 15
	**GEP_low_**	1×10^5	0/3	>20, >20, >20
		1×10^4	0/3	>20, >20, >20
		1×10^3	0/4	>20, >20, >20, >20
**50**	**GEP^high^**	1×10^5	3/3	6, 6, 7
		1×10^4	3/3	8, 10, 10
		1×10^3	4/4	8, 12, 13, 13
	**GEP_low_**	1×10^5	0/3	>20, >20, >20
		1×10^4	0/3	>20, >20, >20
		1×10^3	0/4	>20, >20, >20, >20
**52**	**GEP^high^**	1×10^4	2/2	8, 10
		1×10^3	2/2	9, 14
	**GEP_low_**	1×10^4	0/2	>20, >20
		1×10^3	0/2	>20, >20
**53**	**GEP^high^**	1×10^5	2/2	6, 8
		1×10^4	1/2	6, >20
		1×10^3	0/2	>20, >20
	**GEP_low_**	1×10^5	0/2	>20, >20
		1×10^4	0/2	>20, >20
		1×10^3	0/2	>20, >20

^ Sorted cell populations from clinical HCC specimens.

To demonstrate the *in vivo* self-renewal ability of the cells, xenografts growing from initial inoculation (1° xenografts) were serially transplanted into secondary mouse recipients. Only GEP^high^, but not GEP_low_ cells, could be successfully engrafted and form tumors (Table 3). Note that all mice injected with GEP^high^ form tumors shortly after injection (4-10 weeks). The results indicated that GEP^high^ cells in the tumor mass were able to generate serial xenografts, indicating the *in vivo* self-renewing capacity of GEP-expressing cells.

**Table 3:** Serial transplantation of GEP^high^ cells-derived xenografts

Case no.	Sorted cells^	Tumor incidence	Latency (weeks)
**40**	**GEP^high^**	2/2	4, 5
	**GEP_low_**	0/2	>20, >20
**50**	**GEP^high^**	2/2	8, 10
	**GEP_low_**	0/2	>20, >20
**52**	**GEP^high^**	2/2	9, 9
	**GEP_low_**	0/2	>20, >20
**53**	**GEP^high^**	2/2	4, 5
	**GEP_low_**	0/2	>20, >20

^ Sorted cell populations from GEP^high^ cells-derived xenografts and cell number per injection was 1×10^4.

We characterized the primary (1°) and secondary (2°) xenograft tumors formed by GEP^high^ cells, and revealed low levels of GEP expression (GEP+ population ranging from 0.24- 9.80%) in the tumor bulks (Figure 3B). The fact that transplantation of GEP^high^ cells could generate a heterogeneous tumor mass consisting of both GEP-positive and GEP-negative cells, demonstrated the self-renewal and differentiation ability of GEP-expressing cells in HCC (Figure 3B).

### Association of GEP and β-catenin in clinical HCC specimens

Previously, we showed in HCC cell line models that GEP regulated the expression levels of hepatic CSC markers CD133 and EpCAM, pluripotency-associated signaling molecules β-catenin, SOX2, Oct4 and Nanog at protein levels [17]. Here we studied the association between GEP and these molecules by RT-qPCR. Up-regulation of GEP levels elevated the transcript levels of CD133, β-catenin and SOX2, vice versa for suppression on GEP levels in HCC cell models (Supplementary Figure S1).

**Figure 4:**
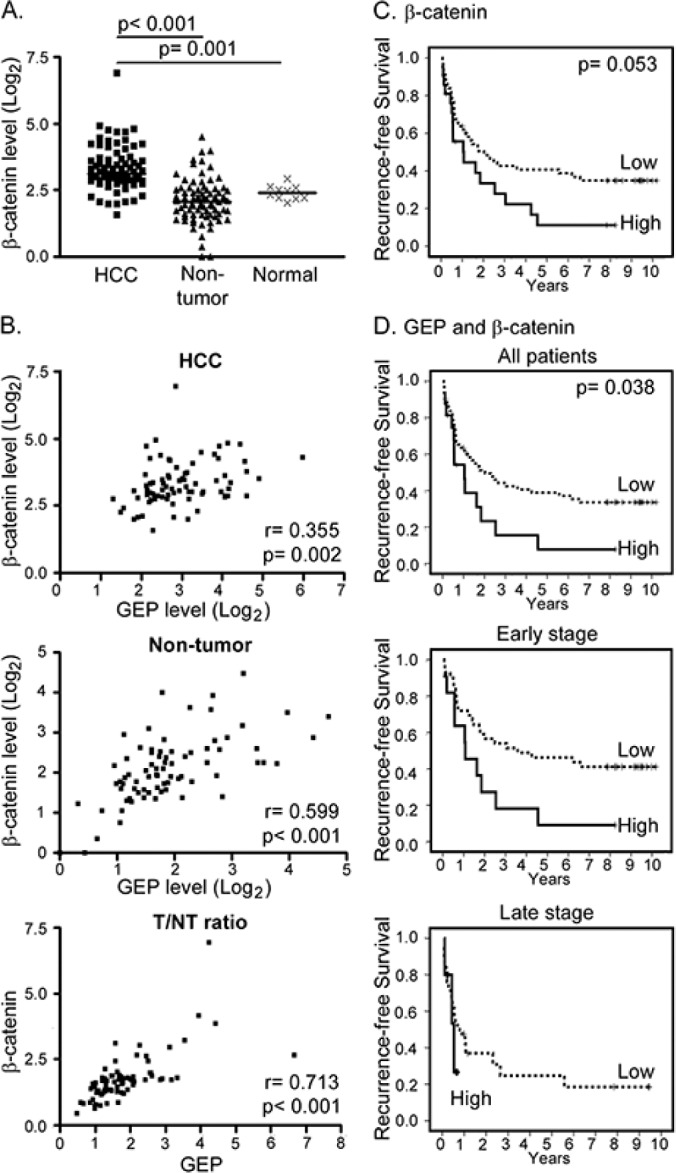
Clinical significance of GEP and β-catenin in HCC clinical specimens **A.** β-catenin transcript level was significantly up-regulated in HCC tumor (HCC, n = 77) compared with the paralleled tumor-adjacent non-tumor liver tissues (non-tumor, n = 77) and normal livers from healthy individuals (normal, n = 10). The lines indicate the median values. **B.** Expression levels of GEP significantly correlated with that of β-catenin in HCC tumor tissues (HCC), and in the paralleled non-tumor tissues. The tumor / non-tumor (T/NT) ratio showed the same trend. **C.** Kaplan–Meier recurrence-free survival plot according to β-catenin levels (log-rank test, p = 0.053). There were 56 patients with low β-catenin expression and 21 patients with high β-catenin expression (median recurrence-free survival of 24.5 months and 12.8 months, respectively). **D.** Patients (n = 77) were segregated into the low expression group (either one or both low in GEP and β-catenin) and the high expression group (both high in GEP and β-catenin). There were 61 patients in the low expression group (median recurrence-free survival, 24.5 months) and 16 patients in the high expression group (median recurrence-free survival, 12.6 months). Patients with high GEP and β-catenin levels were found to have poor recurrence-free survival (log-rank test, p = 0.038). When the patients were segregated into early and late tumor stages, patients with high GEP and β-catenin levels also demonstrated poor recurrence-free survival (log-rank test, p = 0.022).

The transcript levels of GEP, CD133, β-catenin and SOX2 were further examined in clinical specimens. HCC tissues showed elevated expression of β-catenin when compared with their paralleled non-tumor liver tissues (n = 77, p < 0.001) and with the normal livers from healthy individuals (n = 10, p = 0.001) (Figure 4A). SOX2 also showed higher transcript levels in HCC tissue than the non-tumors (n = 30, p = 0.045) (Supplementary Figure S2) with marginal difference on the mean and median levels. However, CD133 transcript levels were higher in non-tumors than HCCs (n = 30, p < 0.001) (Supplementary Figure S2), which might be due to the contamination of CD133+ hematopoietic and endothelial progenitor cells [28, 29]. Thus, the sample size for CD133 and SOX2 was not further increased to parallel the β-catenin full sample set.

Significant correlation between GEP and β-catenin expression was observed in HCC tumor tissues (n = 77, Spearman's ρ correlation coefficient = 0.355, p = 0.002), paralleled non-tumor liver tissues (n = 77, Spearman's ρ correlation coefficient = 0.599, p < 0.001), and tumor/ non-tumor (T/NT) ratio (n = 77, Spearman's ρ correlation coefficient = 0.713; p < 0.001) (Figure 4B).

### β-catenin expression and HCC clinico-pathological features

The association of β-catenin transcript levels with clinico-pathological parameters in HCC patients (n = 77) were further examined. β-catenin levels were significantly associated with Edmondson-Steiner grade (Table 4; p = 0.028). Here we showed that patients with high levels of β-catenin tended to have poor recurrence-free and overall survival, although statistical significance was not reached (log-rank test, p = 0.053 and p = 0.128, respectively) (Figure 4C and Supplementary Figure S3). GEP expression levels have been shown to associate with recurrence [13], therefore β-catenin and GEP levels were assessed together to investigate if this could add incremental prognostic information for HCC patients. The patients were segregated into two groups, the low expression group (low in either one or both GEP and β-catenin levels) and the high expression group (high in both GEP and β-catenin levels). There were 61 patients in the low expression group (median recurrence-free survival, 24.5 months) and 16 patients in the high expression group (median recurrence-free survival, 12.6 months). Notably, high GEP and β-catenin levels were significantly associated with poor recurrence-free survival (log-rank test, p = 0.038). When the patients were segregated into early and late tumor stages, patients with high GEP and β-catenin levels were also found to have poor recurrence-free survival in both subgroups (log-rank test, p = 0.022) (Figure 4D).

**Table 4:** Clinico-pathological features of HCC in relation to β-catenin transcript level

Clinico-pathological features	β-catenin^		*P* value
	Low	High	
**Age**			
Young (≤60)	39	15	0.879
Elderly (>60)	17	6	
**Venous infiltration**			
Absent	29	8	0.284
Present	27	13	
**HBV status**			
Negative for HBsAg	7	5	0.236
Positive for HBsAg	48	16	
**Cellular differentiation (Edmondson-Steiner grade)**			
Well-differentiated	51	15	**0.028**
Poorly-differentiated	5	6	
**Tumor stage (version UICC7)**			
Early stages	36	15	0.555
Late stages	20	6	
**Tumor size**			
Small (≤5cm)	14	4	0.583
Large (>5cm)	42	17	
**Serum AFP level**			
Low (≤400ng/ml)	37	10	0.139
High (>400ng/ml)	19	11	
**Number of tumor nodules**			
Single	39	17	0.321
Multiple (≥2)	17	4	

^ The cutoff for β-catenin was 12.46, which was the mean value of β-catenin transcript level in HCC.

To examine the prediction power for recurrence-free survival, Cox regression analysis was used to compare the GEP and β-catenin levels with tumor stage. By univariable Cox regression analysis, high expression levels of GEP and β-catenin [hazard ratio (HR), 1.9; 95% confidence interval (CI), 1.0–3.7; p = 0.041] and late tumor stage (HR, 1.9; 95% CI, 1.1–3.4; p = 0.028) were significantly associated with poor recurrence-free survival. By multivariable Cox regression analysis, both GEP and β-catenin (HR, 2.2; 95% CI, 1.1–4.2; p = 0.019), and late tumor stage (HR, 2.1; 95% CI, 1.2–3.7; p = 0.014), were independent prognostic factors for recurrence-free survival (Table 5). The above findings demonstrated that high GEP and β-catenin levels significantly influenced the prognosis of HCC patients who underwent curative partial hepatectomy.

**Table 5:** Cox regression analyses for recurrence-free survival on GEP/β-catenin levels and tumor stage

Variables^	n	Univariable analysis			Multivariable analysis		
		HR (95% CI)		*P* value	adjusted HR (95% CI)		*P* value
**GEP/β-catenin**							
Low	61	1.0			1		
High	16	1.9	(1.0-3.7)	0.041	2.2	(1.1-4.2)	0.019
**Tumor stage**							
Early	51	1.0			1		
Late	26	1.9	(1.1-3.4)	0.028	2.1	(1.2-3.7)	0.014

^ GEP and β-catenin levels examined by real-time RT-PCR were modeled as categorical variables in the analyses.

## DISCUSSION

In this study, we have confirmed the role of GEP in hepatic CSCs in HCC clinical specimens. The present work further established GEP as CSC marker and conferred biological function on enrichment of CSC properties including spheroid formation and the ability to form tumor with minimal cell load by GEP positive HCCs directly isolated from clinical specimens. GEP was shown to tightly associate with β-catenin levels in HCC clinical specimens. Furthermore, HCC patients with high GEP and β-catenin expression levels demonstrated poor recurrence-free survival.

The protein levels of GEP and a panel of stem cell related markers in freshly resected HCC specimens were investigated in the present study. The isolated cells were examined for viability, and revealed that the HCC specimens that generated cells with high viability were significantly associated with presence of venous infiltration and poor differentiation, which were aggressive HCC features. GEP levels (by flow cytometry) were positively associated with venous infiltration in the current HCC cohort, which further corroborated our previous report that GEP regulated HCC cell invasion ability and that strong GEP expression (by immunohistochemistry) associated with venous infiltration [11]. Noted that the percentage of GEP+ cells was higher than previously described [17], which may be due to the exclusion of cases with low viability. In fact, we previously reported that GEP protected HCC cells from anoikis-induced apoptosis and GEP levels significantly correlated with the viability of isolated HCCs in single-cell suspension [28]. Therefore, GEP expression empowered the HCC cells to sustain high viability, and vice versa HCCs with high viability showed high GEP expression. Thus, GEP over-expression contributed to the HCC aggressive feature in particular invasion ability and cell viability in detachment which are essential properties for cancer metastasis.

In addition, we showed that β-catenin transcript levels in HCC specimens were significantly associated with cellular differentiation by Edmondson-Steiner grade. Our result echoes the report by other researchers that β-catenin was significantly higher than its non-tumor counterparts, and the increased expression was correlated with Edmondson-Steiner grade [30]. Poorly-differentiated tumors generally exhibited poor prognoses. It was found that poorly-differentiated tumors displayed over-expression of genes that were normally enriched in embryonic stem cells [31]. Moreover, expressions of Oct4, SOX2, Nanog and c-Myc were observed more frequently in poorly-differentiated tumors than in well-differentiated tumors. Such embryonic stem cell signature was also present in poorly differentiated glioblastomas and bladder carcinomas [31]. Here, patients with high levels of β-catenin showed poor recurrence-free survival, though statistical significance had not been reached (p = 0.053), which might probably due to small sample size. However, when combining GEP and β-catenin, it was demonstrated that HCC patients with high GEP and β-catenin levels had significantly poorer recurrence-free survival than the low expression group (log-rank test, *p* = 0.038). The result suggested the effect of GEP and β-catenin by regulating the stemness and invasiveness of tumors for prediction of HCC prognosis.

Activation of β-catenin in HCC induces the expressions of genes encoding for cyclin D1, survivin, c-Myc, EpCAM, and VEGF-A, which contribute to HCC by regulating cell proliferation, survival, metabolism and angiogenesis [32, 33]. Dysregulation of the Wnt/β-catenin was reported to cause transformation of liver stem/progenitor cells, implying a role in self-renewal of hepatic stem cells [34]. Thus, inhibition of β-catenin in a subset of HCC should have significant therapeutic relevance. The small molecule FH535 was shown to prevent Wnt-mediated signaling by suppressing the recruitment of β-catenin co-activators to target gene promoters in HCC cell line model [35]. The expression of cyclin D1 and survivin, the two well-characterized targets of β-catenin, was found to be reduced by FH535 [36]. Besides, it was also illustrated that interferon could inhibit β-catenin signaling by upregulating RanBP3, a nuclear export factor for extruding β-catenin outside the nucleus [37]. However, these molecules were not tested in clinical trials yet, and could be target for therapeutic development in HCC patients.

In addition to GEP and β-catenin, expression of CD90/EpCAM have also been reported in HCC samples, however, with controversial clinical implications [38–41]. CD90 was shown to express in inflamed and normal liver tissues but not exclusively in HCC [38]. In HCC tissues, CD90 was observed on endothelial cells, leukocytes, stroma and also tumor cells [39]. Therefore, additional markers such as CD45 and CD31 would be needed to distinguish the expression of CD90 in hepatic cells from the other cell types. Biological role of EpCAM in carcinogenesis was contentious [40]. EpCAM overexpression was associated with protective or promoting roles in patients with different cancer types [40]. In the current study, EpCAM expression levels varied greatly among individual patients, ranging from 39.2 to 86.3% HCC cells that expressed EpCAM on their surfaces (Figure 1C, EpCAM unsorted cells). Other research group has also reported similar EpCAM expression levels in HCC [41]. Notably, such high proportion of EpCAM+ cells would be doubtful to serve as specific CSC marker. Therefore, additional marker such as AFP would be necessary to co-stain with EpCAM to further delineate the specific EpCAM+ hepatic CSCs [9]. Further effort would be needed to comprehensively examine these markers.

GEP blockage by antibody approach could sensitize HCCs to conventional therapies including chemotherapy [16]. Our group previously demonstrated in HCC cell line models that GEP modulated pluripotency-associated signaling molecules including β-catenin and SOX2 [17]. Here, we showed that GEP was significantly associated with β-catenin and poor clinical outcome in HCC patients. Therefore, GEP might contribute to hepatic CSC properties by regulating pluripotency-associated signaling molecules. Targeting GEP might represent a potential therapeutic strategy to reduce the stemness of CSCs. Further investigations are warranted to investigate the combination therapeutic approach for treatment of the aggressive HCCs.

## MATERIALS AND METHODS

### Clinical specimens

The study protocol was approved by the Institutional Review Board of the University of Hong Kong / Hospital Authority Hong Kong West Cluster (HKU/HA HKW IRB). Patients underwent surgical resection for HCC between 2010 and 2014 at Queen Mary Hospital, Hong Kong, were prospectively recruited with written informed consent. Note that all patients were intended to receive curative resection and therefore extra-hepatic metastasis were only incidental events. Totally 3 out of 90 patients were observed to have extrahepatic diseases at the time of surgery. TOCE/TACE before surgery was performed in 5 out of 90 patients. The mean follow-up period was 19.0 months, while median was 18.2 months. Tumor and adjacent non-tumor liver tissue pairs from HCC patients (n=90) were freshly collected, sliced and digested by type IV collagenase (Sigma-Aldrich, St. Louis, MO). After filtration through 40 micron-cell strainer (BD Biosciences, San Jose, CA) and lysis of red blood cells, cells were counted and assessed for viability by trypan blue staining. Only cases with cell viability ≥ 70% (n = 42) were subject to phenotypic characterization, cell sorting and functional assays in the current study. The present data on CD133, EpCAM, β-catenin, Oct4, Nanog, SOX2 and ABCB5 on sorted cell populations were new data, while part of the GEP data have been reported in our previous studies [17, 18, 42, 43] and the current study has increased sample size to parallel datasets on stem-cell related molecules. Note that each clinical specimen generated only limited number of cells for subsequent experiments, and therefore not enough for comprehensive examination on the full panel of stem cell markers or functional assays. For the stem cell marker investigation, at least 7 clinical specimens were examined. For cancer stem cell properties, at least 3 HCCs were investigated in the functional assays. Details have been described in the result section.

For clinico-pathological association and survival analyses on GEP, β-catenin, CD133 and SOX2, the retrospective clinical cohort were HCC patients who underwent surgical resection for HCC between 2002 and 2005 at Queen Mary Hospital, Hong Kong. All patients were intended to receive curative resection and extra-hepatic metastasis were incidental findings in three patients at the time of surgery. No patient in this cohort received TOCE/TACE before surgery. The mean follow-up period was 107.4 months and median 109.5 months. Tumor and adjacent non-tumor tissue pairs from HCC patients (n = 77) were collected and total RNA was extracted from snap frozen tissue specimens for transcript level analysis using real-time quantitative RT-PCR as previously described [13, 15]. The The β-catenin, CD133 and SOX2 data presented were new data, whereas the GEP data had been extracted from the previous reported cohorts [13, 18] for studying paralleled datasets on β-catenin, CD133 and SOX2.

### Immunofluorescence staining and flow cytometric analysis

For expressions of GEP, β-catenin, Oct4, SOX2, Nanog and ABCB5, cells were permeabilized with ice-cold 0.1% saponin and then incubated with FITC-conjugated mouse anti-human GEP (Versitech Ltd., [14]), Alexa Fluor 647-conjugated mouse anti-human β-catenin, PerCP-Cy5.5-conjugated mouse anti-human Oct4, Alexa Fluor 647-conjugated mouse anti-SOX2, PE-conjugated mouse anti-human Nanog (BD Biosciences), unconjugated goat anti-human ABCB5 (Everest Biotech Ltd, Oxfordshire, UK), or equal amount of corresponding isotype controls. For ABCB5, cells were incubated with NorthernLights (NL) 557-conjugated donkey anti-goat IgG secondary antibody after primary antibody. For CD133 and EpCAM surface expression, cells were stained with APC-conjugated mouse anti-human CD133 (Miltenyi Biotec, Bergisch Gladbach, Germany), APC-conjugated mouse anti-human EpCAM (BD Biosciences) or equal amount of corresponding isotype controls.

### Real-Time quantitative reverse-transcription polymerase chain reaction

Real-time quantitative RT-PCR was performed as described previously [11]. Quantification was performed with the ABI Prism 7700 sequence detection system (Applied Biosystems, Foster City, CA). Primers and probe for GEP have previously been described [11]. Primers and probe were ready-made reagents (Pre-Developed TaqMan Assay Reagents; Applied Biosystems) for CD133, EpCAM, ABCB5, SOX2, Oct4, β-cat and Nanog (catalog no./assay ID: Hs01009250_m1, Hs00901885_m1, Hs00698751_m1, Hs01053049_s1, Hs01895061_u1, Hs00355049_m1 and Hs02387400_g1, respectively), and 18S rRNA internal control. In brief, there were 40 amplification cycles. The sample was regarded as absent for expression when there was no detectable signal up to 40 cycles. The relative amount of the above markers had been normalized with control 18S for RNA amount variation and calibrator for plate-to-plate variation, and the results were presented as the relative fold change.

### Cell sorting

Isolation of GEP-expressing cells was performed by magnetic activated cell sorting (Miltenyi Biotec) according to manufacturer's instructions. Briefly, cells were stained with FITC-conjugated mouse anti-human GEP (Versitech Ltd., [14]), and then incubated with anti-FITC magnetic microbeads (Miltenyi Biotec), prior to magnetic separation. Cells were sorted based on the surface expression of GEP, but not on their intracellular expression, because permeabilization was avoided to keep the cells viable for subsequent functional assays. After cell isolation, sorted cells were assessed for cell viability by trypan blue staining and total cellular GEP levels by flow cytometry using mouse anti-human GEP (R&D systems, Minneapolis, MN) antibody recognizing different GEP epitope. Sorted cells were permeabilised prior to staining so that total GEP expression of the sorted cells could be determined. Cells were then stained with PE-conjugated rabbit anti-mouse secondary antibody. Post-sorting analysis consistently indicated purities >80% with minimal cell death.

### Colony formation assay

Freshly isolated cells were seeded at a density of 1000 cells per 6-well and allowed to grow for a month. Colony formation was assessed by a colorimetric assay using crystal violet (Sigma Aldrich).

### Spheroid formation assay

Cells were seeded to grow as spheroids in non-adherent, serum-free and growth factor-supplemented conditions that favor the proliferation of undifferentiated cells. Disaggregated 1000 cells were cultured in 500 μl of serum-free DMEM/F12 medium (ThermoFisher Scientific Ltd., Waltham, MA), supplemented with 20 ng/ml human recombinant EGF (Sigma Aldrich), 10 ng/ml human recombinant bFGF (ThermoFisher Scientific), 4 μg/ml insulin (Sigma Aldrich), B27 (1: 50; ThermoFisher Scientific). Cells were cultured in ultra-low attachment 24-well plate (Sigma Aldrich). Cells were cultured for 1 month and replenished with 100 μl of supplemented medium every 2-3 days. After 1 month, primary (1°) spheroids were collected, counted, and dissociated to disaggregated cells by trypsin. 1000 disaggregated cells were allowed to grow in DMEM/F12 medium with the above growth factors and supplements for another 1 month for the generation of secondary (2°) spheroids. The 2° spheroids formed after 1 month were then collected, counted and passaged as above mentioned for over 10 generations thereafter.

For epithelial *in vitro* cell differentiation, a few 2° spheroids were dissociated into disaggregated cells and seeded onto 6-well plate in DMEM/F12 medium supplemented with 10% FBS without the above mentioned growth factors and supplements.

### Doxorubicin accumulation

Cells were incubated with or without 0.5 μg/ml doxorubicin for 24h, prior to washing with PBS once. Doxorubicin has intrinsic fluorescence (emission wavelength: 580 nm) and the intracellular doxorubicin level of the cells was detected by flow cytometry at FL2 spectrum as described previously [13, 17, 44].

### *In vivo* tumorigenicity experiments

The study protocol was approved by the Committee on the Use of Live Animals in Teaching and Research at the University of Hong Kong. NOD/SCID mice of 5-8 weeks old were used to assess the *in vivo* tumorigenic potential of the cells. Various cell numbers (1×10^3^ to 1×10^5^) were mixed with Matrigel (BD Biosciences) and inoculated subcutaneously at the dorsal region of the trunk of each animal. Mice were sacrificed between 8 and 20 weeks post-injection, at which tumors were harvested for further investigation. Those mice injected with tumor cells but showed no sign of tumor burden were terminated 4-5 months after cell injection.

### Cell culture and GEP transfection

Human HCC cell lines, Hep3B and HepG2, were purchased from the American Type Culture Collection. Stable transfectants for GEP overexpression was established by transfecting GEP full-length (FL) cDNA into HepG2, a cell line with low endogenous GEP level; while GEP suppression was performed by transfecting GEP shRNA (sh) into Hep3B, a cell line with high endogenous GEP level [11]. All transfectants were maintained in 10% advanced minimum essential media (AMEM) with 0.4 mg/mL of G418.

### Statistical analyzes

All analyzes were performed using the statistical software GraphPad Prism Version 6.00 for Windows (GraphPad Software, CA, USA) or SPSS version 16.0 for Windows (SPSS Inc, Chicago, IL). All *in vitro* data were expressed as mean values ± standard deviation (SD) from at least three independent experiments. Continuous variables were assessed by Spearman correlation and compared between groups by ANOVA (clinical samples) or Student *t* test (*in vitro* models). GEP and β-catenin transcript levels were continuous variables, and the data were modeled as categorical variables in Kaplan–Meier and Cox regression analyses. Mean, median or Youden index (i.e., sensitivity + specificity − 1) had been considered and used to determine the optimal cutoff values for the prediction of 1-year or 3-year recurrence-free survival. The cutoff value for GEP protein expression levels in viable cells isolated from fresh HCC specimens (n = 42) was 6.92% (% of GEP+ cells), which was determined by Youden Index for prediction of 1-yr recurrence-free survival. The cutoff value for GEP transcript levels in archived HCC specimens (n = 77) was 5.83 (relative GEP mRNA levels), which was determined by Youden Index for prediction of 3-yr recurrence-free survival. For β-catenin transcript levels in archived HCC specimens, the cutoff value was 12.46, which was the mean value. The association of GEP, β-catenin and tumor stage (Union Internationale Contre le Cancer version 7, UICC7) with recurrence-free survival was examined by univariable and multivariable Cox proportional hazards regression with a forward stepwise selection procedure. A probability (p) < 0.05 was considered significantly different.

## SUPPLEMENTARY FIGURES AND TABLE


