# Supplementary material for: Hepatic cancer stem cell marker granulin-epithelin precursor and β-catenin expression associate with recurrence in hepatocellular carcinoma
Source: Oncotarget. 2016 Mar 1;7(16):21644–57. doi: 10.18632/oncotarget.7803 (PMC5008312; doi:10.18632/oncotarget.7803)
Supplement: Supplementary file 1 [file oncotarget-07-21644-s001.pdf]

# SUPPLEMENTARY FIGURES AND TABLE

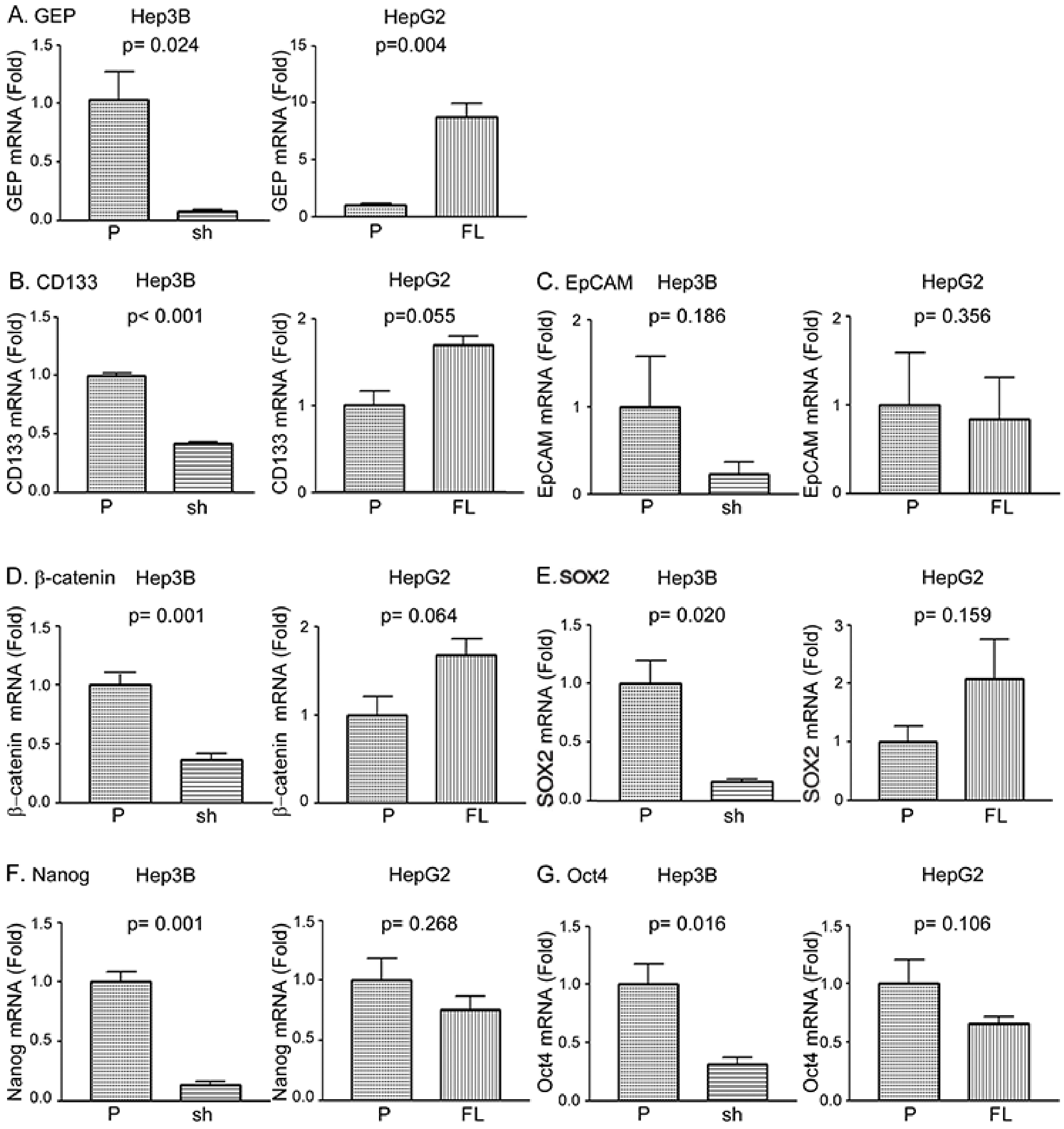


**Supplementary Figure S1: Effect of GEP modulation on the transcript expression levels of hepatic CSC markers and pluripotency-associated genes in HCC cell line models.** GEP expression was modulated by transfection in HCC cell lines Hep3B (P: parental Hep3B cells with high endogenous GEP levels; sh: GEP shRNA transfectants) and HepG2 (P: parental HepG2 cells with low endogenous GEP levels; FL: GEP full-length cDNA transfectants). **A.** GEP transcript level was significantly suppressed in Hep3B sh (left panel) compared to its parental cells P; while significantly over-expressed in HepG2 FL (right panel) compared to its parental cells P. **B–G.** Expressions of CD133, EpCAM, β-catenin, SOX2, Nanog and Oct4 in the GEP transfectants were quantified by RT-qPCR.

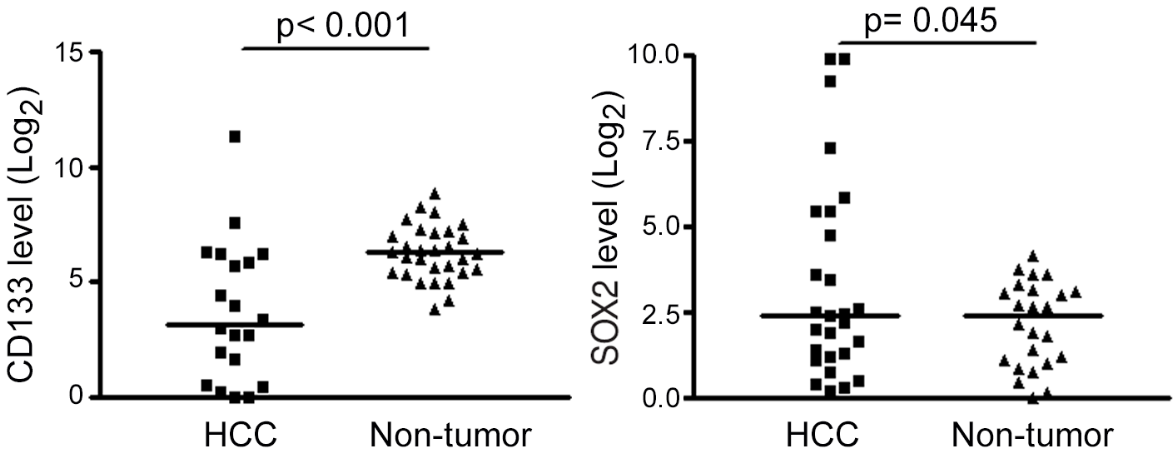


**Supplementary Figure S2: CD133 and SOX2 transcript levels in HCC clinical specimens.** CD133 and SOX2 transcript levels in HCC tumor (HCC, n = 30) and the paralleled tumor-adjacent non-tumor liver tissues (non-tumor, n = 30), with the lines indicating the median values.

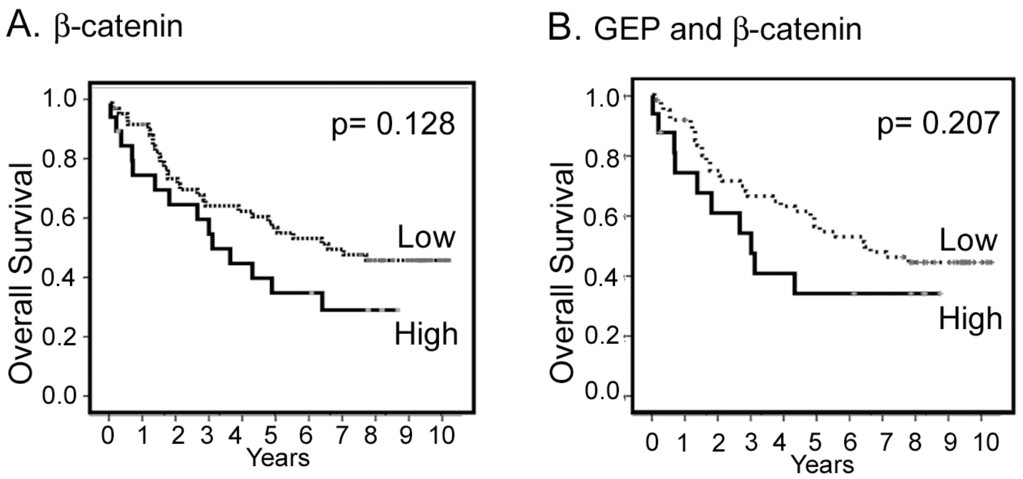


**Supplementary Figure S3: Clinical significance of GEP and β-catenin in HCC clinical specimens. A.** Kaplan–Meier overall survival plot according to β-catenin levels (log-rank test, p = 0.128). There were 56 patients with low β-catenin expression and 21 patients with high β-catenin expression (median overall survival of 85.4 months and 43.9 months, respectively). **B.** Kaplan–Meier overall survival plot according to GEP and β-catenin levels (log-rank test, p = 0.207). Patients (n = 77) were segregated into the low expression group (either one or both low in GEP and β-catenin) and the high expression group (both high in GEP and β-catenin). There were 61 patients in the low expression group (median overall survival, 77.6 months) and 16 patients in the high expression group (median overall survival, 36.1 months).

**Supplementary Table S1: Clinico-pathological features in relation to the viability of HCC cells isolated from clinical specimens**

| Clinico-pathological features | Cell viability^ | | *P* value |
|---|---|---|---|
| | low | high | |
| **Age** | | | |
| Young (≤60) | 24 | 22 | 0.822 |
| Elderly (>60) | 24 | 20 | |
| **Venous infiltration** | | | |
| Absent | 26 | 12 | **0.014** |
| Present | 22 | 30 | |
| **HBV association** | | | |
| Negative for HBsAg | 16 | 10 | 0.320 |
| Positive for HBsAg | 32 | 32 | |
| **Cellular differentiation (Edmondson-Steiner grade)** | | | |
| Well-differentiated | 40 | 26 | **0.008** |
| Poorly-differentiated | 5 | 14 | |
| **Tumor stage (version UICC7)** | | | |
| Early stage | 28 | 20 | 0.386 |
| Late stage | 15 | 16 | |
| **Tumor size** | | | |
| Small (≤5cm) | 19 | 10 | 0.110 |
| Large (>5cm) | 29 | 32 | |
| **Serum AFP level** | | | |
| Low (≤400 ng/ml) | 35 | 21 | **0.025** |
| High (>400 ng/ml) | 13 | 21 | |
| **Number of tumor nodules** | | | |
| Single | 30 | 26 | 0.954 |
| Multiple (≥2) | 18 | 16 | |

^Cell viability was modeled as categorical variable and 70% viability as the cutoff.
